# The comparative accuracy of pooled vs. individual blood culture sampling methods for diagnosis of catheter-related bloodstream infection

**DOI:** 10.1186/s12879-022-07605-x

**Published:** 2022-07-17

**Authors:** Phitphiboon Deawtrakulchai, Surampa Cheawchanwattana, Wantin Sribenjalux, Atibordee Meesing

**Affiliations:** 1 Department of Medicine, Faculty of Medicine, Khon Kaen University, Khon Kaen, 40002 Thailand; 2 Division of Critical Care Medicine, Department of Medicine, Faculty of Medicine, Khon Kaen University, Khon Kaen, 40002 Thailand; 3 Division of Infectious Diseases and Tropical Medicine, Department of Medicine, Faculty of Medicine, Khon Kaen University, Khon Kaen, 40002 Thailand; 4 Research and Diagnostic Center for Emerging Infectious Diseases (RCEID), Khon Kaen University, Khon Kaen, 40002 Thailand

**Keywords:** Catheter-related bloodstream infection (CRBSI), Pooled blood culture, Individual blood culture, Differential time to positivity, Hospital-acquired infections, Infection control

## Abstract

**Background:**

Catheter-related bloodstream infection (CRBSI) is associated with increased morbidity, mortality, and cost of treatment in critically ill patients. A differential time to positivity (DTP) of 120 min or more between blood cultures obtained through the catheter vs. peripheral vein is an indicator of CRBSI with high sensitivity and specificity. However, it is no clear whether pooled sampling would be as efficient as individual sampling in order to reduce costs, contamination, or anemia.

**Methods:**

This was a prospective diagnostic study conducted at the medical ICU and semi-ICU of Khon Kaen University’s Srinagarind Hospital in Thailand from May 2020 to November 2021. Fifty patients with triple-lumen central venous catheters (CVCs) who were clinically suspected of CRBSI were enrolled. 15 mL of blood was drawn through each catheter lumen, 10 mL of which was inoculated into three blood culture bottles, and the remaining 5 mL was pooled into a single bottle. Sensitivity, specificity, accuracy, and time to positivity of the pooled blood cultures were calculated using individual blood cultures as a reference.

**Results:**

Of the 50 patients enrolled, 14 (28%) were diagnosed with CRBSI, 57.9% of whom were infected with gram-negative bacteria as the causative pathogen (57.9%). Extensively drug-resistant (XDR) *Klebsiella pneumoniae* was the most common organism. Sensitivity and specificity of the pooled blood sampling method were 69.23% (95% CI [0.44–0.94]) and 97.3% (95% CI [0.92–1.02]), respectively. The area under the ROC curve (AUC) was 0.83 (95% CI [0.68–0.99]). A paired T-Test to compare time to positivity of the pooled blood bottle and the first positive culture from the individual bottles indicated statistical significance (14.9 and 12.4 h, respectively). The mean difference was 2.5 [0.9–4.1] h, with a 95% CI and a *p*-value of 0.006.

**Conclusion:**

Pooled blood sampling results in a lower sensitivity and longer time to positivity for CRBSI diagnosis in patients with triple-lumen CVCs than individual lumen sampling.

*Trial registration* Retrospectively registered at Thai Clinical Trials Registry. The study was reviewed and approved on 08/03/2022. TCTR identification number is TCTR20220308002

**Supplementary Information:**

The online version contains supplementary material available at 10.1186/s12879-022-07605-x.

## Introduction

Catheter-related bloodstream infection (CRBSI) is associated with increased morbidity, mortality, and cost of treatment in critically ill patients [1–4]. The rates of CRBSI in the intensive care unit (ICU) ranged from 0.6 to 13 per 1000 catheter-days [4, 5]. In the United States, over 80,000 episodes of central venous catheter (CVC)-related bloodstream infection occur in the ICU annually [6]. A report from a surgical ICU in northern Thailand found the incidence of CRBSI to be 1.3 per 1000 catheter-days from 2005 to 2009 [7].

A differential time to positivity (DTP) of 120 min or more (earlier detection of growth in a blood culture obtained through a catheter hub than in a culture of simultaneously drawn peripheral blood) is one indicator that is highly sensitive and specific for CRBSI [6, 8, 9]. It also allows for catheter retention before the definite diagnosis has been made. According to the Infectious Diseases Society of America (IDSA)’s 2009 Clinical Practice Guidelines for the Diagnosis and Management of Intravascular Catheter-Related Infection, when CRBSI is suspected, paired blood cultures should be drawn from the catheter and a peripheral vein before initiation of antimicrobial therapy, and the bottles should be appropriately marked to indicate the site from which the samples were collected [6]. Failing to collect blood samples from some lumens of the catheter can lead to the misdiagnosis of CRBSI episodes [10]. By contrast, drawing blood cultures from all lumens increases laboratory costs and the risk of contamination and may also lead to nosocomial anemia, especially in pediatric patients [11]. Because catheter colonization can occur in any of the lumens [12], pooled blood sampling from all lumens into a single culture bottle might result in higher sensitivity than individual sampling, while simultaneously lowering laboratory costs and the required workload.

This study aims to compare the accuracy of pooled blood sampling from all lumens to that of individual sampling for CRBSI diagnosis.

## Methods

This was a prospective, diagnostic study conducted at the medical ICU and semi-ICU of Khon Kaen University’s Srinagarind Hospital in Thailand from May 2020 to November 2021. Fifty patients with triple-lumen CVC clinically suspected of CRBSI were enrolled. We excluded patients who were under the age of 18, were pregnant, had HIV infection, or in whom any lumen of the CVC was blocked. Blood culture from the CVC and a peripheral blood sample were taken simultaneously before an empirical antibiotic was given. We drew 15 mL of blood from each hub of the CVC aseptically. 10 mL from each sample was inoculated separately into three individual bottles, and then the remaining 5 mL of each sample was pooled into one bottle. The blood volume for each culture bottle was based on IDSA guidelines [13]. Blood culture bottles were incubated in an AutoMate system (BACT/ALERT VIRTUO^®^, BioMerieux, France) at 37° Celcius for 5 days. Identification of all isolates was performed using the VITEK card system (VITEK^®^ 2 Compact, BioMerieux, France). Sensitivity, specificity, and accuracy of the pooled blood culture were calculated using the individual blood cultures as a reference [10]. The time to positivity of each method was compared. Baseline characteristics of the patients, including age, sex, medical conditions, admission diagnosis, and SOFA score, were also recorded.

### Sample size calculation

Sample size calculation was based on a hypothesized sensitivity and specificity of 95% of the pooled blood culture compared to the individual cultures. A pilot study found the sensitivity and specificity of pooled blood culture to diagnose CRBSI caused by bacterial pathogens at Srinagarind Hospital to be 100% and 96.9%, respectively. Based on these factors, we determined that a sample size of at least 45 participants would be necessary.

### Statistical analysis

Baseline characteristics of the patients were reported as number and percentage. Continuous variables were presented as mean with standard deviation (SD). We used 2 × 2 table to calculate the sensitivity, specificity, and accuracy of the pooled blood culture compared to the individual cultures. Subgroup analysis was conducted for bacterial and fungal pathogens. McNemar’s Chi-square test was conducted and area under the receiver operating characteristic curve (AUC) was calculated to demonstrate the performance of the test. All statistical analyses were performed using SPSS for Windows, version 26.0 (SPSS).

### Ethical consideration

Ethical approval was provided by the Khon Kaen University’s Center for Ethics in Human Research in accordance with the Declaration of Helsinki (Number HE621436). Informed consent was obtained from all participants.

## Results

A total of 50 patients who met the inclusion criteria were enrolled into the study. The patients’ median age was 60 years (Interquartile range [IQR] of 53–74 years), 60% were male, and median Sequential Organ Failure Assessment (SOFA) score at ICU admission was 8 (IQR of 5.3–10.0). The most common medical conditions were hypertension and renal failure (34%), followed by diabetes (26%), chronic kidney disease (18%), and malignancy (18%). In half of the cases, the primary diagnosis was pneumonia. Others were CRBSI, septic shock, and urinary tract infection (Table 1).

**Table 1:** Baseline characteristics of all 50 patients

Variables	N = 50
Mean Age, years (SD)	59.4 (19.1)
Median Age, years (IQR)	60 (53, 74)
Male, n (%)	30 (60)
Sites of enrollment, n (%)	
MICU1	10 (20)
MICU2	22 (44)
MIMCU	18 (36)
Vital signs at the time of blood culture taking, median (IQR)	
Systolic blood pressure, mmHg	110.5 (96.5–130)
Diastolic blood pressure, mmHg	60 (50.5–78)
Mean arterial pressure, mmHg	79 (65.5–95.3)
Heart rate, beats per minute	110 (88.5–125)
Respiratory rate, breaths per minute	24 (20–28)
Body temperature, Celsius degree	37.8 (36.8–38.5)
SOFA score, median (IQR)	8 (5.25–10)
Primary diagnosis, n (%)	
Pneumonia	25 (50)
CRBSI	14 (28)
Septic shock	13 (26)
Urinary tract infection	4 (8)
Skin and musculoskeletal infection	4 (8)
Intraabdominal infection	3 (6)
Hepatobiliary tract infection	2 (4)
Infective endocarditis	1 (2)
Comorbidities, n (%)	
Hypertension	17 (34)
AKI	17 (34)
Diabetes mellitus	13 (26)
CKD	9 (18)
Malignancy	9 (18)
Received immunosuppressive drugs	9 (18)
COPD/Chronic lung disease	5 (10)
Current steroid usage	5 (10)
Liver cirrhosis	4 (8)
Regular hemodialysis	3 (6)
CAPD	2 (4)

*AKI* acute kidney injury, *CAPD* continuous ambulatory peritoneal dialysis, *CKD* chronic kidney disease, *COPD* chronic obstructive pulmonary disease, *CRBSI* catheter-related bloodstream infection, *IQR* interquartile range, *MICU* medical intensive care unit, *MIMCU* medical intermediate care unit, *SD* standard deviation, *SOFA* Sequential Organ Failure Assessment

Fourteen patients (28%) in the study were diagnosed with CRBSI based on a DTP of 120 min or more. In three cases, there was coagulase negative staphylococci and in another there was *Bacillus* spp. in the peripherical blood, which was considered contamination. Cultures from both the CVC and peripheral site in the remaining cases were negative. There was no significant difference in duration of catheterization before blood culture collection between those with infected and non-infected CVCs (171.4 vs. 205.7 h, *p*-value 0.57). Most CVCs in the study were inserted into right internal jugular vein, followed by left internal jugular vein, and right femoral vein. We could not find any association between site of catheterization and rate of infection (*p*-value 0.67; Table 2).

**Table 2:** Comparison of duration of catheterization before blood culture collection and site of CVC between CRBSI and non-CRBSI cases

Variables	CRBSI (N = 14)	non-CRBSI (N = 36)	*p*-value	95% CI
Mean duration of catheterization before blood culture collection, hours (SD)*	171.4 (99.5)	205.7 (207.9)	0.57	− 83.4–150.1
Sites of CVCs**				
Right internal jugular vein, n (%)	9 (25)	27 (75)	0.67	–
Left internal jugular vein, n (%)	2 (40)	3 (60)		
Right femoral vein, n (%)	2 (50)	2 (50)		
Left subclavian vein, n (%)	0 (0)	2 (100)		
Right femoral vein, n (%)	1 (33.3)	2 (66.7)		

CRBSI, catheter-related bloodstream infection; CVC, central venous catheter; SD, standard deviation

^*^Independent T-test for testing the difference of duration of catheterization before blood culture collection between positive and negative blood culture cases

^**^Fisher-Freeman-Halton Exact Test used to determine the difference in CVC infection rate by site of insertion

**Fig. 1:**
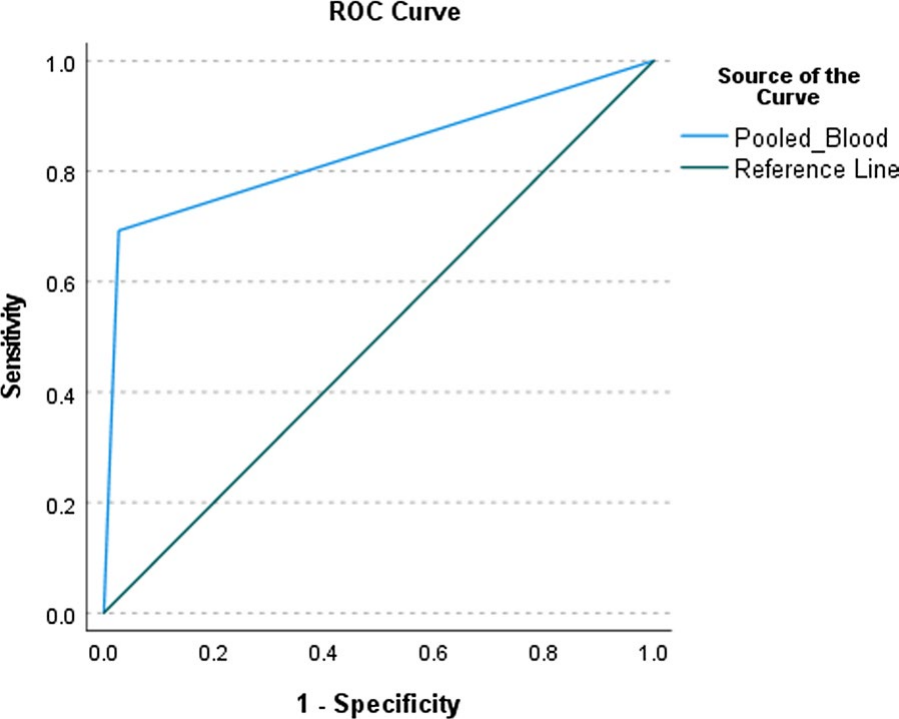
AUC of the diagnostic test using pooled and separate blood sampling from each port of the central line

Three of 14 patients were infected by multiple microorganisms, resulting in a total of 19 identified microbial species, 11 (57.9%) of which were gram-negative bacteria, five were gram-positive, and the remaining were *Candida* spp. The most common bacterial pathogens found were extensively drug-resistant (XDR) *Klebsiella pneumoniae.* Thirteen of the 14 patients met the diagnosis criteria based on individual blood sampling (71.4% from the proximal, 50% from the middle, and 42.9% from the distal port), compared to only 10 based on pooled sampling. The sensitivity, specificity, positive predictive value, and negative predictive value of pooled blood sampling to diagnose of CRBSI were 69.23% (95% CI [0.44–0.94]), 97.3% (95% CI [0.92–1. 02]), 90% (95% CI [0.71–1.09]), and 90% (95% CI [0.80–0.99]), respectively (Table 3). The AUC calculated to assess the performance of the pooled blood sampling was 0.83 (95% CI [0.68–0.99]; Fig. 1).

**Table 3:** Diagnostic performance of pooled blood sampling for CRBSI diagnosis when using individual blood culture as a gold standard

Culture profile		Individual blood	
		Positive culture, N (%)	Negative culture, N (%)
Pooled blood	Positive culture, N (%)	9 (69.2)	1 (2.7)
	Negative culture, N (%)	4 (30.8)	36 (97.3)
*p*-value		< 0.01	
Sensitivity (%, 95% CI)		69.2 (0.44–0.94)	
Specificity (%, 95% CI)		97.3 (0.92–1.02)	
Positive predictive value (%, 95% CI)		90 (0.71–1.09)	
Negative predictive value (%, 95% CI)		90 (0.80–0.99)	
Positive likelihood ratio (95% CI)		25.62 (3.58–177.63)	
Negative likelihood ratio (95% CI)		0.32 (0.15–0.76)

In seven of the nine patients with positive culture from both pooled and individual blood bottles, pathogens were discovered in the individual samples before the pooled samples. A paired T-Test to compare time to positivity of the pooled blood bottle and the first positive culture from the individual bottles indicated statistical significance (14.9 vs. 12.4 h, respectively). The mean difference with 95% CI was 2.5 [0.9–4.1] h, with a *p*-value of 0.006. A detailed comparison of time to positivity of the blood culture results from all 14 CRSBI patients is available in the supplemental material (Additional file 1: Table S1).

## Discussion

This was a prospective, diagnostic trial comparing the accuracy of CRBSI diagnosis between two blood sampling methods. To our knowledge, this was the first such study conducted in Thailand. Fourteen of the 50 patients with suspected CRBSI had positive blood cultures from the peripheral site and one or more positive cultures from the CVC. Gram-negative bacteria were the predominant pathogens, which is consistent with the epidemiologic data in Thailand. However, our study differed in that we discovered a high predominance of XDR pathogens [7]. Data from National Antimicrobial Resistance Surveillance center, Thailand (NARST) show an exponential rise in the percentage of carbapenem-resistant Enterobacterales from 2015 to 2020 [14]. Similar findings have been reported in Japan and other parts of Southeast Asia, reflecting the ICU crisis in the region [15].

Pooled blood sampling misidentified 4 of the 13 cultures that were found positive by individual blood sampling and resulted in significantly increased in time to positivity. This suggests that pooled blood sampling might not be a good alternative method for diagnosing CRBSI, contradicting the findings of a previous study by Herrera-Guerra et al. [16]. One explanation for these findings may be the type of catheter used. Herrera-Guerra et al. used both double and triple lumen catheters, but only patients with triple lumen catheters were enrolled into our study. Another may be blood volume. Herrera-Guerra et al. drew 6 mL of blood from each lumen, inoculating 4.5 mL into individual blood culture bottles and the remaining 1.5 mL into a pooled blood bottle, while our study compared 10 mL in individual bottles to 5 mL from three lumens in the pooled blood bottle [16]. Sensitivity of blood culture is related to blood sample volume [17–20]. In this study, if bacterial colonization occurred in only one lumen of the CVC, pooled blood bottles would yield 5 mL of infected blood compared to 10 mL in individual blood bottles, resulting in lower sensitivity and increased time to positivity. In most cases with positive culture based on the individual sampling method, only one of three bottles tested positive. Single lumen colonization is common in CRBSI [10], which may explain these results. Blood-broth ratio is one factor that affects the positive rate of blood culture, with a ratio of 1:5 to 1:10 considered optimal for growth [21]. Pooled blood bottles can have a suboptimal blood-broth ratio, causing slower bacterial growth.

Two of the three cases of *Candida* spp. found by individual blood sampling were negative using pooled sampling. Larger inoculum size is also positively associated with *Candida* spp. detection using automated blood culture systems, especially in cases of *C. glabrata*, which has a longer mean time to growth detection [22]. Although a previous study found that DTP yields good sensitivity and specificity for catheter-related candidemia diagnosis, pooled blood sampling is not recommended [23]. Other studies have recommended against using DTP to diagnose CRBSI from *Candida* spp. Due to slower growth rate of fungi, the standard cut-off of 120 min might not be optimum [24–26]. In one case, BSI was detected only by pooled blood sampling, but the recovered pathogen was *B. pseudomallei*, which rarely causes CRBSI [2]. Although few nosocomial infections caused by *B. pseudomallei* have been reported, most can be explained by direct inoculation of the micro-organism during medical procedures (e.g., wound dressing and bronchoscopy) [27, 28].

There were some limitations to this study. First, the results cannot be applied to pediatric populations, as pediatric blood culture bottles are adapted to contain a smaller blood volume. For neonates, 1 mL of blood is adequate for detection of bacteremia [19]. The broth-blood ratio of pooled blood cultures in pediatric bottles also differs from that in adult bottles. Second, although DTP can be applied in both triple-lumen and double-lumen catheters [6], and single lumen colonization also occurs in double-lumen catheters, our results may not apply in cases with double-lumen catheters. This is because pooled samples from triple-lumen CVCs will be more diluted in cases of single lumen colonization. Further studies should be conducted to examine the accuracy of this test in other types of CVC. Another limitation was the small sample size. Only fourteen cases of confirmed CRBSI were studied. Despite these limitations, this study attained valuable evidence that pooled blood culture has a lower sensitivity and requires a longer time for CRBSI diagnosis compared to individual blood culture due to the smaller volume of blood that is drawn.

Pooled blood sampling has a lower sensitivity and higher time to positivity for CRBSI diagnosis in patients with triple-lumen CVCs. Laboratory costs, workload, and potential side effects (e.g., nosocomial anemia) should be considered when deciding whether to use this method. To maximize sensitivity, we encourage individual rather than pooled blood sampling.

## Supplementary Information

**Additional file 1: Table S1.** Blood culture results of all 14 CRSBI patients and comparison of time to positivity between pooled and individual blood sampling bottles.

## Data Availability

The data that underlie the results reported in this study are available from the corresponding author on reasonable request.
