# Supplementary material for: The comparative accuracy of pooled vs. individual blood culture sampling methods for diagnosis of catheter-related bloodstream infection
Source: BMC Infect Dis. 2022 Jul 17;22:622. doi: 10.1186/s12879-022-07605-x (PMC9290260; doi:10.1186/s12879-022-07605-x)
Supplement: Supplementary file 1 — Additional file 1: Table S1. Blood culture results of all 14 CRSBI patients and comparison of time to positivity between pooled and individual blood sampling bottles. [file 12879_2022_7605_MOESM1_ESM.docx]

Supplemental Data

Table S1 Blood culture results of all 14 CRSBI patients and comparison of time to positivity between pooled and individual blood sampling bottles

| No. | Central line sites | Duration of usage, Hr. | Blood culture result and TTP (hours) | | | | | | | | | |
| --- | --- | --- | --- | --- | --- | --- | --- | --- | --- | --- | --- | --- |
|  |  |  | Pooled blood | | Proximal port | | Middle port | | Distal port | | Peripheral blood | |
|  |  |  | Pathogen | TTP | Pathogen | TTP | Pathogen | TTP | Pathogen | TTP | Pathogen | TTP |
| 1 | Right IJV | 109 | *Candida albicans* | 30.8 | *Candida albicans* | 30.5 | *Candida albicans* | 31.7 | *Candida albicans* | 28.7 | *Candida albicans* | 33.8 |
| 2 | Right IJV | 101 | *Klebsiella pneumoniae (XDR)*  *Enterococcus faecalis*  Coagulase-negative staphylococci | 10.3 | *Klebsiella pneumoniae (XDR)*  *Enterococcus faecalis*  Coagulase-negative staphylococci | 10.1 | NG |  | NG | - | *Klebsiella pneumoniae (XDR)*  *Enterococcus faecalis*  Coagulase-negative staphylococci | 13.5 |
| 3 | Right IJV | 120 | *Burkholderia pseudomallei* | 65.5 | NG | *-* | NG |  | NG | - | *Burkholderia pseudomallei* | 72.5 |
| 4 | Right FV | 420 | *Acinetobacter baumannii* (CRAB) | 7.2 | NG | *-* | *Acinetobacter baumannii* (CRAB) | 6.8 | NG | - | *Acinetobacter baumannii* (CRAB) | 10.4 |
| 5 | Right IJV | 213 | NG |  | *Candida albicans* | 28.6 | NG | - | NG | - | *Candida albicans* | 33.6 |
| 6 | Right IJV | 123 | *Enterococcus faecium Stenotrophomonas maltophilia* | 14.7 | NG |  | *Enterococcus faecium Stenotrophomonas maltophilia* | 14.4 | *Candida grabata* | 3.1 | *Enterococcus faecium Stenotrophomonas maltophilia* | 17.5 |
| 7 | Right IJV | 168 | *E. coli*  *Klebsiella pneumoniae (XDR)* | 15.7 | *E. coli*  *Klebsiella pneumoniae (XDR)* | 13 | *E. coli*  *Klebsiella pneumoniae (XDR)* | 11.5 | *E. coli*  *Klebsiella pneumoniae (XDR)* | 14.8 | *E. coli*  *Klebsiella pneumoniae (XDR)* | 18.2 |
| 8 | Right FV | 156 | *Staphylococcus hemolyticus* | 18.6 | NG | *-* | NG | *-* | *Staphylococcus hemolyticus* | 12.9 | *Staphylococcus hemolyticus* | 20.8 |
| 9 | Right IJV | 256 | NG | *-* | *Enterococcus faecium* | 13.2 | *Enterococcus faecium* | 13.2 | NG | *-* | *Enterococcus faecium* | 16.6 |
| 10 | Right IJV | 167 | NG | *-* | *Klebsiella pneumoniae (XDR)* | 27.4 | NG | *-* | NG | *-* | *Klebsiella pneumoniae (XDR)* | 35.1 |
| 11 | Left IJV | 50 | NG | *-* | *Burkhholderia cepacia* | 31.8 | NG | *-* | NG | *-* | *Burkhholderia cepacia* | 40.4 |
| 12 | Left IJV | 75 | *Klebsiella pneumoniae* (XDR) | 10.3 | *Klebsiella pneumoniae* (XDR) | 8.6 | *Klebsiella pneumoniae* (XDR) | 9.4 | *Klebsiella pneumoniae* (XDR) | 5.4 | *Klebsiella pneumoniae* (XDR) | 13.7 |
| 13 | Right FV | 267 | *Klebsiella pneumoniae* (XDR) | 9.3 | *Klebsiella pneumoniae* (XDR) | 7.3 | *Klebsiella pneumoniae* (XDR) | 8.2 | *Klebsiella pneumoniae* (XDR) | 10.3 | *Klebsiella pneumoniae* (XDR) | 11.5 |
| 14 | Right IJV | 184 | *Elizabethkingia meningoseptica* | 17.5 | *Elizab*e*thkingia meningoseptica* | 14.5 | NG | *-* | NG | *-* | *Elizab*e*thkingia meningoseptica* | 20.3 |
| TTP analysis*  (N=9) | | | Pooled blood bottle, mean (hours) +/-SD  14.9 ± 7.1 | | First positive culture from the individual bottles, mean (hours) +/-SD  12.4 ± 7.0 | | | | | | *p*-value = 0.006,  mean difference (hours) [95%CI]: 2.5 [0.9-4.1] | |

*Paired T-Test between TTP of pooled blood bottle and the first positive culture from individual blood bottles

CRAB, Carbapenem-resistant Acinetobacter baumannii; FV, femoral vein; IJV, internal jugular vein; NG, no growth; TTP, time to positivity; XDR, extensively drug-resistant
